# Optimizing cefazolin dosing for central nervous system infections: insights from population pharmacokinetics and Monte Carlo simulations

**DOI:** 10.1128/aac.01857-24

**Published:** 2025-05-20

**Authors:** C.Tyler Pitcock, David S. Burgess, Katie B. Olney

**Affiliations:** 1 Department of Pharmacy, Duke University Hospital 22957, Durham, North Carolina, USA; 2 Department of Pharmacy Practice and Science, University of Kentucky College of Pharmacy 15511, Lexington, Kentucky, USA; 3 Department of Pharmacy Services, University of Kentucky HealthCare 177468, Lexington, Kentucky, USA; University of California San Francisco, San Francisco, California, USA

**Keywords:** pharmacokinetic, pharmacodynamic, cefazolin, CNS

## Abstract

Recent studies suggest that cefazolin represents an appropriate treatment option for methicillin-susceptible *Staphylococcus aureus* (MSSA) central nervous system (CNS) infections. Monte Carlo simulations were used to evaluate specific cefazolin dosing regimens to identify the optimal dosing strategy to ensure adequate probability of target attainment for treatment of MSSA CNS infections.

## INTRODUCTION

Cefazolin, a first-generation cephalosporin, has historically been believed to have poor blood-brain barrier penetration and thus considered inadequate for treatment of central nervous system (CNS) infections, including meningitis. This belief is largely based on literature evaluating cephalothin, a parenteral first-generation cephalosporin, which has since been removed from the market, and limited case series evaluating cefazolin CNS penetration in patients with uninflamed meninges (1, 2).

On the contrary, recent case reports have demonstrated the ability of cefazolin to achieve adequate cerebrospinal fluid (CSF) and brain tissue concentrations well above the typical minimum inhibitory concentration (MIC) for methicillin-susceptible *Staphylococcus aureus* (MSSA), suggesting its potential as a viable option for CNS infections (3–6). As such, we sought to identify the optimal cefazolin dosing regimens necessary to achieve the pharmacodynamic (PD) efficacy target for MSSA CNS infections.

Cefazolin plasma and CSF pharmacokinetic (PK) parameters were obtained from a previously published study, which included 15 patients with external ventricular drains (EVDs) receiving cefazolin for prophylaxis (4). All patients received cefazolin 2 g IV every 8 h. CSF and serum cefazolin concentrations were drawn at a median of 2.5 days [interquartile range (IQR), 1.5–7] from the first cefazolin dose. Serum and CSF samples were collected at various time points during administration. The majority of patients were female (80%) with a median age of 56 years (IQR, 51–60), median weight of 72 kg (IQR, 65–89), and median creatinine clearance of 115 mL/min (IQR, 84–174). Pertinent PK parameters, including median CSF half-life of 6.5 h (IQR, 5.5–13.1) and median CSF:serum AUC ratio of 6.7% (IQR, 3.9–11.3), were used to estimate CNS penetration. Additional baseline demographics of the included patients are shown in Table 1. Oracle Crystal Ball software was used to perform 10,000-subject Monte Carlo simulations. Simulations were performed for cefazolin regimens ranging from 6 to 12 g/day administered intermittently every 4–8 h, over 30 min, or as a continuous infusion (CI) over 24 h. CNS penetration was used in lieu of protein binding because only unbound drug concentration can cross through the blood-brain barrier (7). The probability of achieving the PD target of 40% *f*T > MIC for intermittent infusions and 100% fT > MIC and 100% fT > 4× MIC for CI was analyzed for MICs of 0.5, 1, and 2 mg/L. These MICs were selected based on MIC_50/90_ and epidemiologic cutoff values from historical breakpoint standards (3, 4). The PD target of 40% *f*T > MIC was selected for intermittent regimens based on prior correlation with efficacy for treatment of *Staphylococcus aureus* infections (8). The PD targets of 100% fT > MIC and 100% fT > 4× MIC were evaluated for dosing regimens administered as a continuous infusion based on literature suggesting higher targets for critically ill patients to improve clinical outcomes (9, 10). Overall, at least 90% probability of target attainment (PTA) was considered adequate.

**TABLE 1:** Baseline patient demographics and pharmacokinetic parameters used for Monte Carlo simulation^*a*^

Parameter (4)	All patients *N* = 15
Age, years^*b*^	56 (51–60)
Gender, female^*c*^	12 (80)
Weight, kg^*b*^	72 (65–89)
Height, cm^*b*^	168 (164–173)
CSF half-life, hours^*b*^	6.5 (5.5–13.1)
CSF: serum AUC ratio, %^*b*^	6.7 (3.9–11.3)
Volume of distribution, L^*b*^	31.4 (13.8–49.8)
Clearance, L/h^*b*^	6.6 (4.1–9.7)
Creatinine clearance, mL/min^*b*^	115 (84–174)

^*a*^ CSF, cerebrospinal fluid; AUC, area under the curve.

^*b*^ Median (IQR).

^*c*^ Number (%).

Analysis of CNS exposures for each of the evaluated regimens is displayed in Tables 2 to 4. The standard cefazolin dosing regimen of 2 g administered as an intermittent infusion every 8 h did not achieve ≥90% PTA for any MIC evaluated. All other regimens achieved ≥90% PTA only when the MIC remained ≤0.5 mg/L. Only 10 and 12 g continuous infusion regimens of cefazolin were capable of achieving ≥90% PTA when the MIC was 1 mg/L. No continuous infusion regimen achieved ≥90% PTA when using the PD target of 100% fT >4× MIC. When comparing the same total daily dose administered as an intermittent versus continuous infusion, continuous infusions were more likely to achieve PD target attainment, particularly when the cefazolin MIC exceeded 0.5 mg/L.

**TABLE 2:** Probability of target attainment of 40% fT > MIC for intermittent infusions^*a*^^,^^*b*^

Dosing regimen	MIC = 0.5	MIC = 1	MIC = 2
2 grams every 8 hours	86%	67%	39%
2 grams every 6 hours	**91%**	76%	49%
2 grams every 4 hours	**96%**	84%	58%

^*a*^ MIC, minimum inhibitory concentration.

^*b*^ Bold indicates the probability of target attainment ≥ 90%.

**TABLE 3:** Probability of target attainment of 100% *f*T >MIC for continuous infusions^*a*^,^*b*^

Dosing regimen	MIC = 0.5	MIC = 1	MIC = 2
6 g every 24 h	**95%**	82%	56%
8 g every 24 h	**98%**	89%	68%
10 g every 24 h	**99%**	**93%**	76%
12 g every 24 h	**99%**	**95%**	82%

^*a*^ MIC, minimum inhibitory concentration.

^*b*^ Bold indicates the probability of target attainment ≥ 90%.

**TABLE 4:** Probability of target attainment of 100% fT > 4× MIC for continuous infusions^*a*^

Dosing regimen	4× > MIC of 0.5	4× > MIC of 1	4× > MIC of 2
6 g every 24 h	56%	27%	9%
8 g every 24 h	68%	39%	15%
10 g every 24 h	76%	48%	21%
12 g every 24 h	82%	56%	27%

^*a*^ MIC, minimum inhibitory concentration.

To the authors’ knowledge, this is the first study analyzing the probability of target attainment for cefazolin in the CNS using patient population PK parameters. These simulations suggest that higher than standard doses of cefazolin are necessary to achieve adequate probability of target attainment, specifically for isolates with increased MICs (≥0.5 mg/L). In 2012, the Clinical and Laboratory Standards Institute recommended the elimination of *Staphylococcus* breakpoints for beta-lactams other than penicillin, oxacillin, ceftaroline, and cefoxitin. This recommendation was based on the ability to infer susceptibilities for other beta-lactams from penicillin, oxacillin, or cefoxitin (11). Although our results indicate standard doses may achieve PD targets for MSSA CNS infections when the cefazolin MIC remains ≤0.5 mg/L (MIC_50_), the inability to perform susceptibility testing for cefazolin necessitates the use of higher than standard doses to achieve PD target attainment with cefazolin MICs of 1 and 2 mg/L (MIC_90_ and epidemiologic cutoff values from historical breakpoint standards, respectively).

Our study has several limitations. First, PK parameters were evaluated from cefazolin concentrations measured directly from patients with EVDs receiving cefazolin for prophylaxis, and we assume that such concentrations reflect concentrations within the CSF compartment. As such, inflammation may not be comparable to that in patients with ventriculitis or meningitis. In the setting of increased meningeal inflammation, enhanced distribution of cefazolin across the blood-brain barrier would be anticipated (12). Second, while cefazolin is generally well-tolerated at standard doses, there are limited data regarding its safety and tolerability at higher doses, which should be taken into consideration (13). Additionally, other PK parameters, such as serum albumin, were not reported within the original study. Given that cefazolin is ~80% protein bound, fluctuations or aberrations in albumin may impact overall drug clearance and subsequent PTA (14). Lastly, the possibility of blaZ gene production by MSSA must be considered when treating any patient with deep-seated infections, including CNS infections. MSSA isolates carrying blaZ are prone to the “cefazolin inoculum effect,” a phenomenon describing elevated cefazolin MICs in the setting of increased organism load (15); however, results of previous studies are mixed, and the clinical impact remains unclear (16–20).

These findings underscore the inadequacy of standard intermittent cefazolin regimens in achieving desired PD targets for MSSA CNS infections. Higher total daily doses or CI dosing strategies are necessary to achieve satisfactory PD targets for MSSA CNS infections with cefazolin. Ultimately, further clinical studies evaluating the safety and efficacy of cefazolin for CNS infections caused by MSSA should be explored.
